# Aspartate Is A Determinant of TNF‐α Biogenesis in Sub‐Saharan Africa: Insight Into the Pathogenesis of Noncommunicable Diseases

**DOI:** 10.1096/fj.202500738R

**Published:** 2025-06-09

**Authors:** Stephen W. Bickler, David M. Cauvi, Jack A. Gilbert, Romeo C. Ignacio, Emmanuel A. Ameh, Robert T. Schooley, Emilia V. Noormahomed

**Affiliations:** ^1^ Department of Surgery, Division of Pediatric Surgery UC San Diego San Diego California USA; ^2^ Division of Pediatric Surgery Rady Children's Hospital San Diego USA; ^3^ University of California, San Diego, Scripps Institution of Oceanography La Jolla California USA; ^4^ Department of Pediatrics UC San Diego San Diego California USA; ^5^ Division of Pediatric Surgery, Department of Surgery National Hospital Abuja Nigeria; ^6^ Department of Medicine UC San Diego San Diego California USA; ^7^ Department of Microbiology, Faculty of Medicine Universidade Eduardo Mondlane (UEM) Maputo Mozambique

**Keywords:** aspartate, inflammation, malate–aspartate shuttle, noncommunicable diseases, sub‐Saharan Africa, tumor necrosis factor‐α, urbanization

## Abstract

Urbanization in sub‐Saharan Africa is characterized by a shift toward a proinflammatory state and rising rates of noncommunicable diseases. The biological mechanism(s) linking changes in environmental factors to health and disease are incompletely understood. We propose a TNF‐hypersecreting phenotype described in rheumatoid arthritis patients also has a role in the inflammatory response differences observed between rural and urban populations living in Africa. The mechanism involves insufficient mitochondrial aspartate production, failed NAD^+^ regeneration, ER membrane expansion, and enhanced biogenesis of TNF‐α. Supporting data show serum and stool aspartate levels decline with urbanization, and TNF‐α production inversely correlates with stool aspartate levels across a spectrum of socioeconomic development. These findings suggest a new hypothesis for the inflammatory differences between rural and urban populations and their role in noncommunicable diseases like atherosclerosis.

## Graphical abstract

Proposed change in TNF‐α biogenesis with urbanization in sub‐Saharan Africa. Higher levels of aspartate in the rural population result in a NAD^+^ rich environment that favors stabilization of the endoplasmic reticulum (ER) membrane and suppressing TNF‐α. With urbanization, aspartate and NAD^+^ levels decrease, resulting in the expansion of ER membranes and increased production of the TNF‐α.

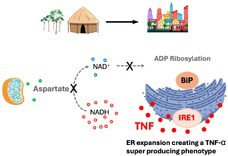

**Abbreviations**
ADP: Adenosine diphosphate
BiP: Binding immunoglobulin protein
ER: Endoplasmic reticulum
GOT2: Glutamic oxaloacetic transaminase 2
GRP78: Glucose‐regulated protein 78
GTP: Guanosine‐5′‐triphosphate
HSPA5: Heat shock protein family A (Hsp70) member 5
IFN‐g: Interferon gamma
IL‐6: Interleukin 6
IRE1: Inositol requiring enzyme‐1
LPS: Lipopolysaccharide
NAD: Nicotinamide adenine dinucleotide
NCDs: Noncommunicable diseases
TCA cycle: Tricarboxylic acid cycle
TNF‐α: Tumor necrosis factor‐alpha

## 1 Introduction

Urbanization in low‐income countries represents a critical inflection point in disease epidemiology, with infectious diseases predominating in rural areas and urban populations taking on a profile of noncommunicable diseases (NCDs) [1, 2, 3]. This epidemiological transition is also evident at the global level, where NCDs have rapidly increased over the past 40 years [4]. Of the 55 million global deaths reported in 2019, 74% were due to NCDs, principally cardiovascular diseases, cancers, chronic respiratory diseases, and diabetes [5]. With no end in sight to the global epidemic of NCDs, there is a critical need to better understand the biological and environmental pressures influencing NCD prevalence. We believe this may be possible by studying the biological changes that occur with urbanization in sub‐Saharan Africa, where urbanization is occurring rapidly and there are often extreme differences in the rural and urban environments.

One of the most enigmatic questions surrounding the rural–urban transition is: “What mechanism(s) underlay the markedly different inflammatory responses in the rural and urban populations?” In the rural environment, inflammation is integral to the immunological response to chronic and recurrent infections. In contrast, in the urban environment, an exaggerated inflammatory response is important in the pathogenesis of NCDs—most notably atherosclerosis and autoimmune diseases. This immunological paradigm is aptly illustrated by ex vivo whole blood immune stimulation tests, where urban dwellers have higher production of the proinflammatory cytokines, tumor necrosis factor‐alpha (TNF‐α), interferon gamma (IFN‐g), and interleukin 6 (IL‐6), and rural populations have a relative suppression of cytokine secretion [6]. Regarding the latter, there must be anti‐inflammatory inhibitory mechanisms, allowing for a response that is “just right” [7].

Recently, aspartate was shown to have a critical role in the biogenesis of TNF‐α [8]. Aspartate is a precursor for asparagine, isoleucine, methionine, lysine, threonine, pyrimidines, NAD, and pantothenate; a nitrogen donor for arginine and purine synthesis; and an important metabolic effector controlling the interconversion of C_3_ and C_4_ intermediates [9]. In patients with rheumatoid arthritis, a shortage of T‐cell mitochondrial aspartate disrupted the regeneration of the metabolic cofactor nicotinamide adenine dinucleotide (NAD), causing ADP deribosylation of the endoplasmic reticulum (ER) sensor GRP78/BiP (HSPA5), the master regulator of the ER stress response [10, 11]. As a result, ribosome‐rich ER membranes expanded, creating a hypersecreting TNF‐α phenotype. The transfer of intact mitochondria into T cells and supplementation of exogenous aspartate rescued the mitochondria‐instructed expansion of ER membranes and suppressed TNF‐α release and rheumatoid tissue inflammation. Furthermore, healthy T cells responded to glutamic oxaloacetic transaminase 2 (GOT2) loss of function with increased formation of ER membranes and upregulation of the ER stress gene signature. Collectively, these observations delineate a mechanistic connection between insufficient mitochondrial aspartate production, failed regeneration of the electron acceptor NAD^+^, expansion of the ER membrane system, and hypersecretion of TNF‐α.

*Aspartate is a key determinant of TNF‐α biogenesis in rural and urban populations living in sub‐Saharan Africa*.

**FIGURE 1:**
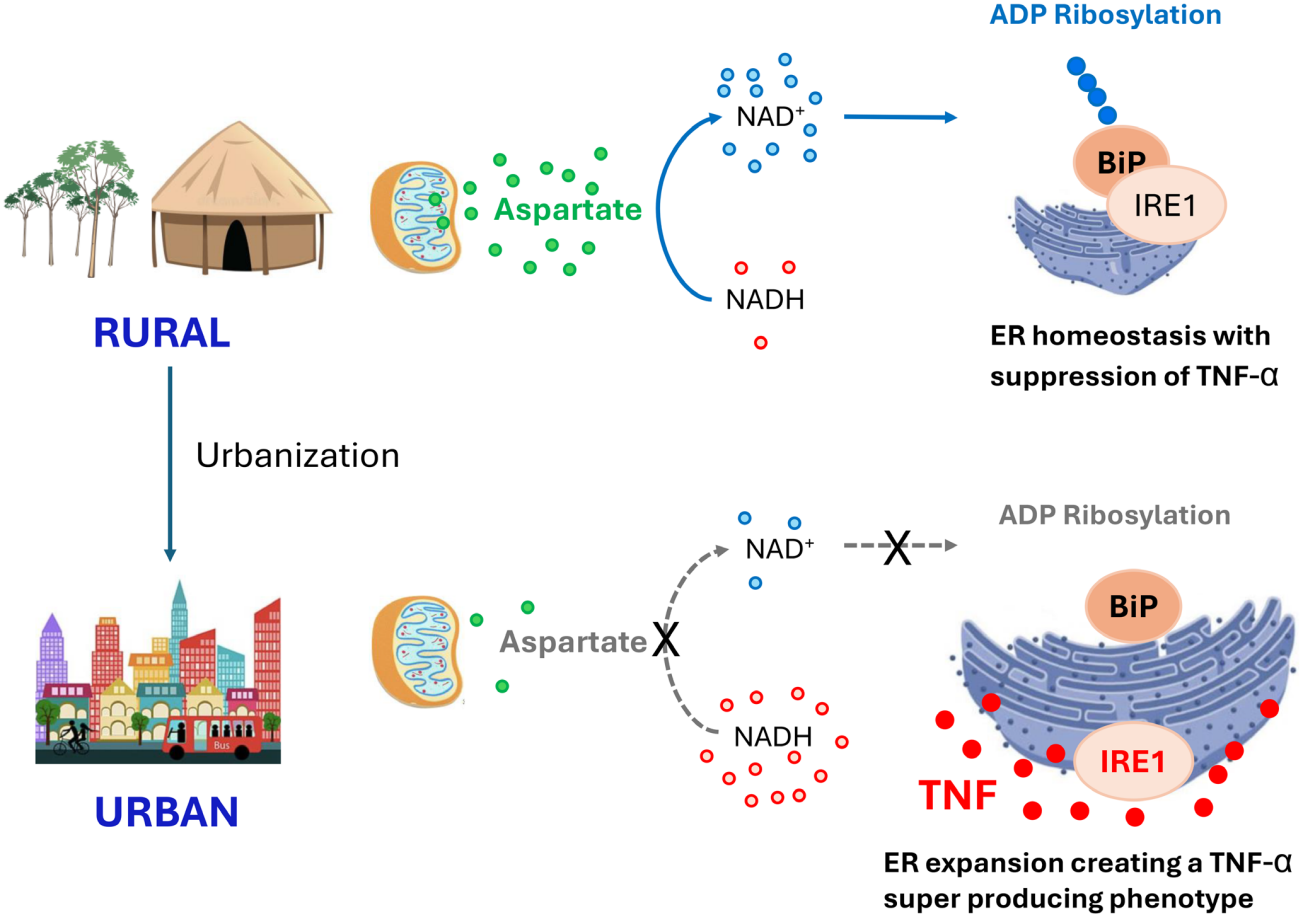
Proposed relationship between aspartate and TNF‐α biogenesis in rural and urban sub‐Saharan Africa. Higher levels of aspartate in the rural population result in a ^+^NAD^+^‐rich environment which favors ADP ribosylation of the BiP/IRE1 complex and stabilization of the endoplasmic reticulum (ER) membrane. With urbanization, aspartate and NAD^+^ levels decrease, resulting in expansion of ER membranes and increased production of the TNF‐α. Adapted from figure 7j, Wu et al. [8].

**FIGURE 2:**
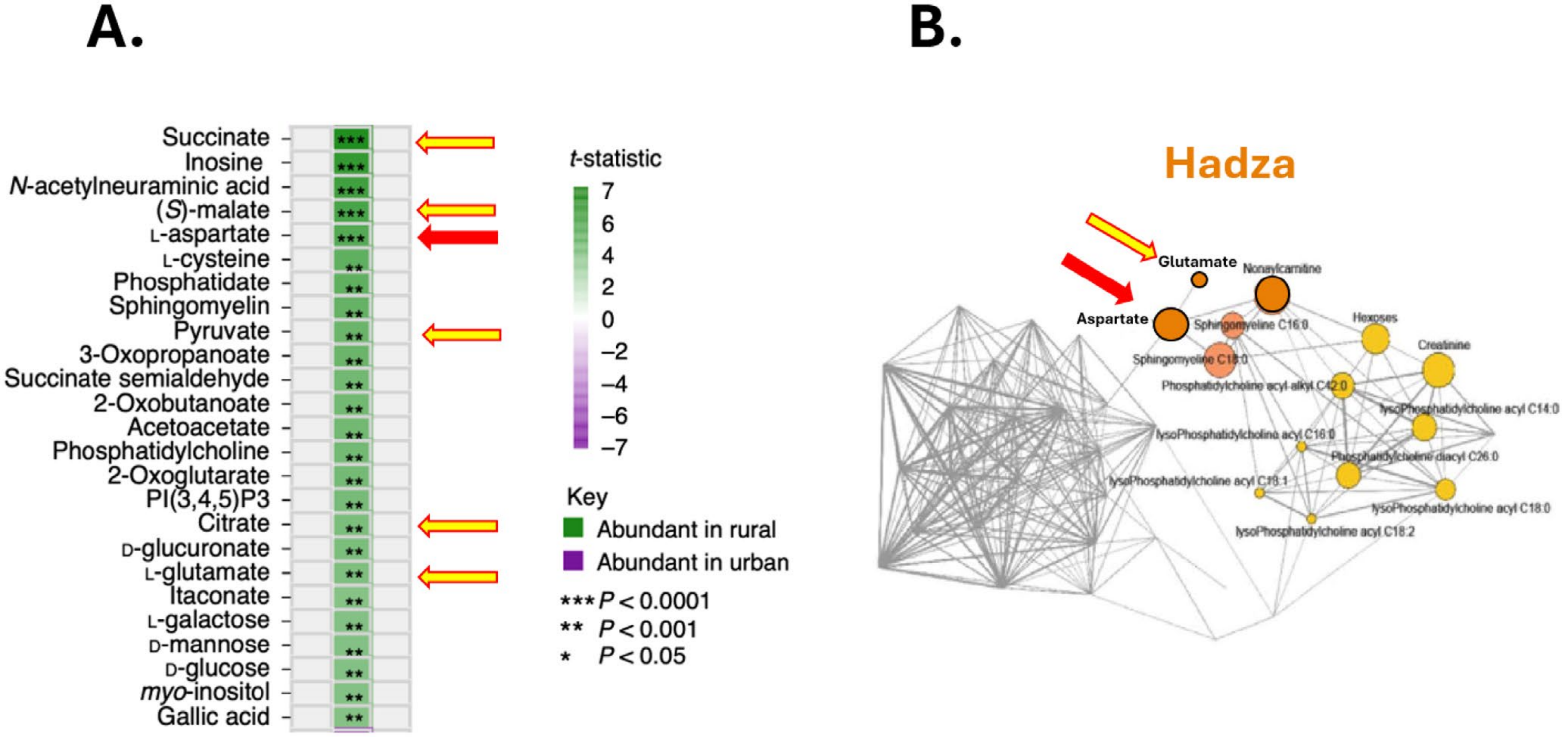
The effect of urbanization on the serum and fecal metabolome. Arrows indicate aspartate (red) and metabolic intermediates that are either part of or closely related to the TCA cycle (yellow). (A) Serum metabolite abundance of rural and urban living Tanzanians. Data from figure 4a, Temba et al. [6]. (B) Wiggum plot of the fecal metabolome in the Hadza people, a traditional rural and foraging society in Tanzania. Each node represents a fecal metabolite, and its dimension is proportional to the overabundance relative to background. Data from figure 4, Turroni et al. [12].

Here, we propose the aspartate–TNF‐α biogenesis mechanism that results in a TNF‐α hypersecreting phenotype in rheumatoid arthritis patients also explains in part the inflammatory differences between rural and urban areas in sub‐Saharan Africa (Figure 1). The following observations support our hypothesis:

1. *Urbanization in sub‐Saharan Africa is associated with decreased serum and fecal levels of aspartate*. Using untargeted metabolomics, Temba et al. [6] identified 348 serum metabolites significantly elevated in a rural compared to the urban cohort in Tanzania, with another 157 metabolites elevated in the urban group. Some of the most significant differences were intermediates either part of or closely linked to the TCA cycle (succinate, inosine, (*S*)‐malate, l‐aspartate, pyruvate, and citrate) (Figure 2A). Studies on the stool of the same Tanzanian subjects found elevated levels of l‐aspartate in the rural compared to the urban cohort [13]. l‐Aspartate was also more prevalent in the stool of the Hadza hunter–gatherers of Tanzania than in Italians [12] (Figure 2B). Stool l‐aspartate levels were not elevated in a comparative study of Nigeria's rural (Bassa) and urban populations. However, the authors speculated that this might be due to the Bassa cohort being further along the urbanization scale than the Hadza people.
2. *Fecal aspartate levels are inversely related to TNF production in an immune stimulation model across a spectrum of socioeconomic development*. Stražar et al. [13] examined the relationship between intestinal microbial composition and whole‐blood cytokine response in a rural and urban population living in Tanzania and a Dutch cohort. The Tanzanian cohort was from rural and urban settings, representing a range of lifestyles, diets, and microbial exposures. Compared to the urban Tanzanians and Europeans, the rural Tanzanian population had lower cytokine responses (TNF‐α and IFN‐g) overall. Three stool compounds (l‐aspartate, fumarate, and *N*‐acetylornithine) in the arginine biosynthesis pathway (KEGG pathway KO0020) were correlated with TNF response to *Candida albicans* stimulation.

The observation that urbanization in sub‐Saharan Africa is associated with changes in serum aspartate evokes several questions. Foremost is (1) the source of the increased aspartate levels in the rural population and (2) the biological consequences of the reduced aspartate levels in the urban setting. Answering these questions could provide insight into how the environment shapes the inflammatory response and the pathogenesis of NCDs.

## 2 Source of the Increased Aspartate Levels in the Rural Population

Serum aspartate levels are influenced by diet, type, and quantity of gastrointestinal microbiota and the amount generated from the tricarboxylic acid (TCA) cycle. Aspartate is abundant in protein‐rich foods such as meat, fish, eggs, and plant sources like soybeans (legumes) and asparagus [14]. Conversely, low‐protein diets or malnutrition may result in reduced serum aspartate levels. Intestinal bacteria can influence serum aspartate levels by modulating amino acid metabolism and absorption in the gut [15, 16]. Dysbiosis, or an imbalance in gut microbiota, may impair this process, leading to altered serum aspartate levels. Species such as *Bifidobacterium* and *Streptococcus* are known to produce aspartate through their metabolic activities [17, 18].

With respect to endogenous production, aspartate is primarily synthesized within the mitochondrial matrix, where it is produced from the tricarboxylic acid (TCA) cycle intermediate oxaloacetate through a transamination reaction [9]. Conditions that enhance TCA cycle activity, such as increased energy demands or enhanced glucose metabolism, can drive higher production of oxaloacetate and, consequently, aspartate. The observation that TCA intermediates (succinate, pyruvate, citrate, and l‐glutamate) are higher in rural compared to urban Tanzanians (Figure 2A) raises the possibility that the alterations in serum aspartate levels are part of broader changes in the TCA cycle.

## 3 Biological Consequences of the Reduced Aspartate Levels in the Urban Population

Based on our hypothesis, low aspartate in the urban population would result in a TNF‐α hypersecreting phenotype. As TNF‐α is a key proinflammatory cytokine involved in immune regulation, apoptosis, and cellular metabolism, an exaggerated TNF‐α response could lead to chronic inflammation, tissue damage, and metabolic dysregulation [19]. This effect is evident in conditions such as autoimmune diseases, where sustained TNF‐α activation drives joint destruction in rheumatoid arthritis or intestinal damage in inflammatory bowel disease [20]. Prolonged TNF‐α elevation can impair mitochondrial function, disrupt oxidative phosphorylation, and promote insulin resistance, contributing to metabolic disorders like type 2 diabetes. TNF‐α has also been implicated in the development and progression of atherosclerosis, affecting multiple stages of the disease, starting with the initial phases of endothelial dysfunction and continuing to the later stages of plaque formation and destabilization [21, 22].

Lower aspartate levels in urban populations also suggest possible changes in redox pathways. Aspartate has a central role in the malate–aspartate shuttle (Borst cycle), which enables NADH transport between the cytosol and mitochondrial matrix [23]. When considered alongside research indicating OXPHOS gene enrichment with urbanization [6, 24, 25], a broader pattern emerges, where aspartate might be part of a larger system designed to maintain redox balance in the face of environmental challenges.

## 4 Testing the Hypothesis

Our hypothesis could be tested using the ex vivo immune stimulation model used by Temba et al. [6] to show the differences in the inflammatory response between rural and urban areas of Tanzania. Whole blood is stimulated with agents like lipopolysaccharide (LPS) for monocytes/macrophages and ionomycin for T cells to induce TNF‐α production. Based on our hypothesis, we would expect the higher TNF‐α production in the urban population to be associated with lower aspartate, greater NADH, and an expanded ER volume. Flow cytometry would be ideal for these studies as it can simultaneously provide cell‐specific information on cytokine production, ER volume, mitochondrial function, and NAD^+^/NADH ratios. Other studies will be needed to determine whether the source of the elevated serum aspartate in the rural population is from the diet, gastrointestinal bacteria, or the TCA cycle. Quantified metagenomic sequencing could be used to characterize the diversity and functional potential of the gut microbiome [26] and isotope tracing using labeled substrates such as [13C]‐glucose could provide information on fundamental changes in the TCA cycle [27].

## 5 Conclusions

In this hypothesis article, we suggest a mechanism that produces a TNF‐α hypersecreting state in rheumatoid arthritis patients, which may also explain the exaggerated inflammatory response in urban compared to rural areas of sub‐Saharan Africa. This mechanism connects insufficient mitochondrial aspartate production, failed regeneration of the electron acceptor NAD^+^, and expansion of the ER membrane system to hypersecretion of TNF‐α. To support our hypothesis, we summarize data from the literature showing serum and stool aspartate levels decrease with urbanization, and TNF production in an ex vivo immunity model is inversely related to stool aspartate levels. Together, these observations create a new testable hypothesis for the difference in the inflammatory response between rural and urban populations in sub‐Saharan Africa and for NCDs in high‐income countries in which inflammation has an important role (e.g., atherosclerosis and autoimmune diseases). The rural–urban paradigm in sub‐Saharan Africa offers an important opportunity to investigate how environmental factors influence serum aspartate levels and its role in determining health and disease.

## Author Contributions

Conceptualization and original draft preparation: **Stephen W. Bickler**. Writing – review and editing: **David M. Cauvi**, **Jack A. Gilbert**, **Romeo C. Ignacio**, **Emmanuel A. Ameh**, **Robert T. Schooley**, **Emilia V. Noormahomed**. All authors have read and agreed to the published version of the manuscript.

## Disclosure

The authors have nothing to report.

## Conflicts of Interest

The authors declare no conflicts of interest.

## Data Availability

No new data.
